# Investigating the Role of Cortical Microglia in a Mouse Model of Viral Infection-Induced Seizures

**DOI:** 10.1523/ENEURO.0374-25.2026

**Published:** 2026-02-24

**Authors:** Lakshmini Balachandar, Lauren Buxton, Ireland Kearns, Matthew F. Stefanic, Laura A. Bell, Ana Beatriz DePaula-Silva, Karen S. Wilcox

**Affiliations:** Department of Pharmacology and Toxicology, University of Utah, Salt Lake City, Utah 84112

**Keywords:** calcium imaging, cortex, cytokines, microglia, purinergic signaling, Theiler's murine encephalomyelitis virus

## Abstract

Microglia, resident immune sentinels in the brain, are crucial in responding to tissue damage, infection, damage signals like purines (ATP/ADP), and clearing cellular debris. It is currently unknown how microglial reactivity progresses and contributes to seizure development following Theiler's murine encephalomyelitis virus (TMEV) infection. Previously, it has been demonstrated that purinergic signaling in microglia is disrupted in the hippocampus of TMEV-infected mice. However, whether reactive cortical microglia also exhibit changes in purinergic signaling, cytokine levels, and purinergic receptors is unknown. Thus, we seek to evaluate region-based differences in microglial reactivity in the TMEV model. We employed a custom triple transgenic mouse line expressing tdTomato and GCaMP6f under a CX3CR1 Cre promoter and exogenously applied ATP/ADP to acute brain slice preparations from TMEV-infected mice and controls of either sex. Interestingly and in contrast to what is observed in the hippocampus, we found that despite microglial reactivity in the cortex, microglia can respond to purinergic damage signals and engage calcium signaling pathways, comparable to PBS controls. Using a cytokine panel, we also found that proinflammatory cytokine levels (TNF-α, IL-1α, and IFN-γ) are brain region dependent in mice infected with TMEV. Using RNAscope FISH, we observed increases in expression of purinergic receptors responsible for microglial motility (P2Y_12_R) and inflammation (P2X_7_R) in the cortex. Collectively our results suggest that following TMEV infection, microglial response to novel damage signals, as well as the production of proinflammatory cytokines, varies as a function of the brain region.

## Significance Statement

Microglia, innate immune brain cells, respond to tissue damage, infection, and have elevated calcium transients in epilepsy. Viral infection-induced seizures likely originate in the hippocampus and, over time, begin to secondarily generalize to the cortex. Despite recent advances, there is a major gap in understanding the region-specific role of reactive microglia in seizure development. Using a mouse model of viral infection-induced epilepsy, we found that cortical microglia retain their ability to respond to novel purinergic damage cues in acute brain slice preparations, despite being reactive, and have enhanced purinergic receptor and cytokine expression as compared with saline controls. These findings pave the way for future investigation on the impact of regional gene expression changes in reactive microglia in seizure generation.

## Introduction

The Theiler's murine encephalomyelitis virus (TMEV) mouse model of infection-induced temporal lobe epilepsy (TLE) is an invaluable tool in epilepsy research for investigating novel therapeutic approaches to prevent viral infection-induced seizures in patients (DePaula-Silva et al., 2021). Mice (C57Bl6/J) infected with the Daniel's strain of TMEV display both spontaneous and handling-induced seizures during the acute phase following infection (3–8 dpi; Libbey et al., 2008; Patel et al., 2017). They develop significant neuronal loss; persistent gliosis across several brain regions, i.e., hippocampus, cortex, and limbic system (Stewart et al., 2010a; Loewen et al., 2016; Bell et al., 2020); and dramatic increases in expression of proconvulsant cytokines and reactive oxygen species (Kirkman et al., 2010; Umpierre et al., 2014; Bhuyan et al., 2015; Patel et al., 2017). Several weeks later, seizure thresholds are reduced, and majority of animals that demonstrated acute seizures develop TLE and behavioral comorbidities (Stewart et al., 2010a,b; Umpierre et al., 2014; Bröer et al., 2016). Seizures likely originate in the hippocampus and, over the course of infection, begin to secondarily generalize to the cortex (Patel et al., 2017).

Microglia are resident immune cells in the central nervous system (CNS) that play a crucial role in responding to tissue damage and infection (Illes et al., 2020; Qin et al., 2023). Microglial calcium signaling has been recognized as a dynamic mediator of responses to neuronal activity and CNS injury, with important implications for epilepsy pathophysiology. During homeostatic conditions, microglia rarely exhibit spontaneous calcium activity (Brawek and Garaschuk, 2013; Pozner et al., 2015; Umpierre et al., 2020). Microglial calcium transients are closely correlated with shifts in neuronal network activity and are enhanced during hyperexcitable environments like kainate-induced SE, indicating that abnormal neuronal excitation drives microglial calcium elevations in epileptogenesis (Umpierre et al., 2020). The increase in frequency of calcium transients is also accompanied by directional microglial process movement (Eichhoff et al., 2011; Brawek et al., 2014; Pozner et al., 2015; Umpierre et al., 2020). Targeted disruption of purinergic P2Y_6_ receptors attenuates microglial calcium signaling and associated process activation during early epileptogenesis, elucidating the role of specific calcium-linked receptor pathways in microglial functional alterations in epilepsy models (Umpierre et al., 2024). These calcium dynamics likely intersect with broader microglial reactive states including phagocytosis, chemotaxis, and cytokine release that contribute to synaptic remodeling and network hyperexcitability in epilepsy.

Seizures also induce microglial interactions with neuronal somata and dendrites, including via purinergic receptors. Microglial migration and chemotaxis are heavily dependent on purinergic receptors including the P2Y_12_ receptor, which is predominantly expressed in microglia (Dissing-Olesen et al., 2014; Eyo et al., 2014; Gómez Morillas et al., 2021). Reactive microglia in the TMEV model have been reported to have significant gene expression changes including downregulation of P2Y_12_R (DePaula-Silva et al., 2019; Wallis et al., 2024). They also exhibit morphofunctional changes, differential expression of surveillance genes governing damage signal recognition, and purinergic receptors including P2X_7_R and P2Y_12_R (Jimenez-Pacheco et al., 2013; Lively and Schlichter, 2018; DePaula-Silva et al., 2019; Hammond et al., 2019). Functional upregulation of P2X_7_R has been observed in microglia in vivo in human and rodent TLE models and, following lipopolysaccharide injections, led to high levels of ATP and upregulation of proinflammatory cytokines (Choi et al., 2007; Jimenez-Pacheco et al., 2013; Di Virgilio et al., 2017). Additionally, P2X_7_R antagonism led to reduction in spontaneous seizures and gliosis in TLE (Jimenez-Pacheco et al., 2016). Following CNS infection, reactive microglia along with infiltrating macrophages contribute to seizure activity via the release of cytokines (Cusick et al., 2013; Wilcox and Vezzani, 2014); reactive microglia also serve as a major source of TNF-α in TLE (Henning et al., 2023).

Recently, during acute TMEV infection, reactive hippocampal microglia had disrupted calcium signaling responses to local purinergic application and exhibited dampened motility toward laser-burn damage, potentially due to downregulated hippocampal P2Y_12_R expression (Wallis et al., 2024). Since the cortex is involved in generalized seizures in the TMEV model (Patel et al., 2017), whether reactive cortical microglial calcium signaling and responses to damage cues could lead to production of proinflammatory cytokines, changes in cytoskeletal receptors, and eventual contribution to seizure propagation is to be investigated.

In the present study, we found that reactive cortical microglia, unlike hippocampal reactive microglia in the same model, can surprisingly respond to novel purinergic damage cues (ATP and ADP) comparable to PBS controls, during the peak period of TMEV infection (5 dpi). We observed increased levels of inflammatory markers like TNF-α, IL-1α, IFN-γ, and IFN-β, among others, in the cortices of TMEV mice, as compared with PBS controls, which were further increased in the hippocampus. Using RNAscope FISH, we also observed a significantly higher expression of P2Y_12_R, P2X_7_R, and TNF-α in cortical microglia post-TMEV infection. Given that many studies have indicated that P2Y_12_R expression is generally decreased in reactive microglia in the acute seizure phase, this was also a novel finding. Overall, cortical microglia, during the acute peak period of TMEV infection, retain their ability to respond to novel purinergic damage cues, despite being reactive, and have enhanced purinergic receptor and cytokine expression as compared with PBS controls. Following TMEV infection, there is reduced expression of cytokine and chemokine profiles in the cortex as compared with the hippocampus. These findings pave the way for future investigation on the impact of regional gene expression changes in reactive microglia in seizure generation.

## Materials and Methods

**Figure 1.**
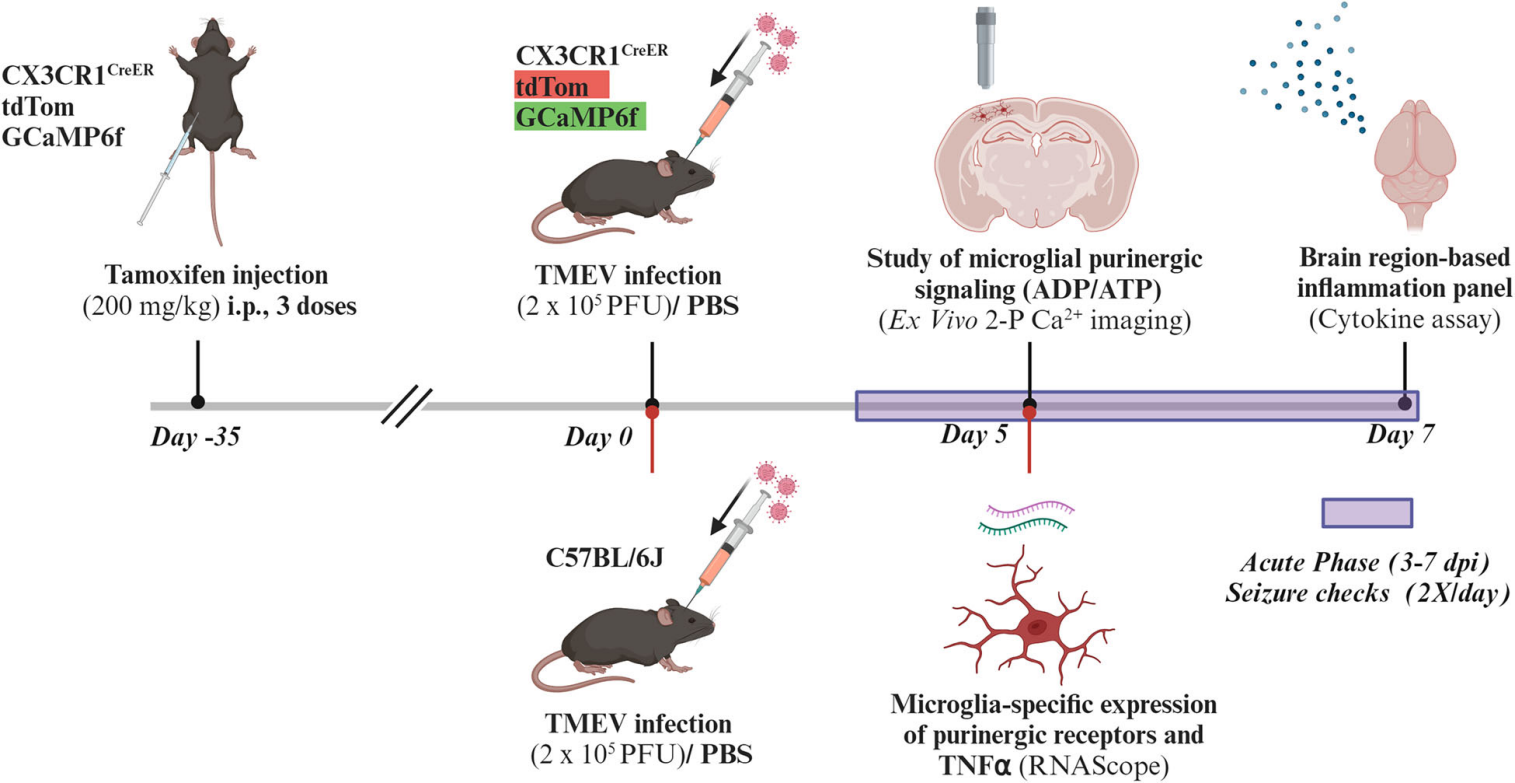
Timeline of TAM administration, TMEV infection in mice, seizure monitoring, and cellular, molecular, and calcium imaging experiments performed in this study. Mice heterozygous for CX3CR1-tdTomato-GCaMP6f were injected with TAM (intraperitoneally) to induce expression of tdTom and GCaMP6f. After at least 35 d to allow for macrophages to turn over and not express the transgenes, mice were injected intracranially with TMEV or PBS and were monitored twice a day from 3 to 7 d for seizures. Acute brain slices containing the parietal or somatosensory cortex and hippocampus were obtained from mice at 5 dpi for 2-P calcium imaging. At 7 dpi in a separate cohort, mice brains were collected for the cytokine assay. Bottom panel of timeline, C57BL/6J (naive) mice were injected with TMEV or PBS intracranially and were monitored twice a day from 3 to 5 d for seizures. At 5 dpi, mice brains were prepared for RNAscope analysis. Figure created using Biorender.

The experimental procedures performed as part of this study were carried out in compliance with the National Institutes of Health Guide for the Care and Use of Laboratory Animals (https://grants.nih.gov/grants/olaw/guide-for-the-care-and-use-of-laboratory-animals) and ARRIVE guidelines (Percie du Sert et al., 2020) and approved by the Institutional Animal Care and Use Committee. The timeline of experiments and various techniques employed in this study are shown in Figure 1.

### Animals

Mice were housed in standard cages, provided with food (Teklad Global Soy Protein-Free Extruded Rodent Diet catalog #2920X; Harlan Laboratories) and water *ad libitum* in a 12–12 h light/dark cycle. All experiments were randomized and performed in both female and male mice. Custom triple transgenic mice heterozygous for CX3CR1, tdTomato (tdTom), and Lck-GCaMP6f were generated from Jackson (JAX) lab mice lines (020940, 007914, and 029626) at our vivarium. Expressions of all transgenes were confirmed by PCR. These transgenic mice were used for experiments involving acute brain slices and cytokine assays. For RNAscope experiments, wild-type C57BL/6J mice (JAX labs 000664) were used. After arrival, the mice were acclimatized to our animal facility and diet for at least 1 week before experiments. The data for the experiments from both sexes of mice were pooled, since there is no suggestion of sex-specific differences in seizure behavior in the TMEV model (Patel et al., 2017).

### Tamoxifen-induced recombination

Transgenic mice (older than 5 weeks of age) were administered with three doses of tamoxifen (TAM; Sigma-Aldrich T5648) dissolved in peanut oil (20 mg/ml; 200 mg/kg, i.p., every 48 h) to induce Cre-mediated gene expression of tdTomato and Lck-GCaMP6f. The use of CX3CR1 creERT2/+ mice meant that infiltrating macrophages would also express GCaMP6f and tdTom thereby making it difficult to distinguish from resident microglia (Cusick et al., 2013). Therefore, subsequent treatments were conducted at least 35 d after the last TAM injection, so that infiltrating macrophages would not display the TAM-induced fluorophores (Parkhurst et al., 2013).

### TMEV infection and seizure monitoring

Mice were injected with 20 µl of either 2.5 × 10^5^ PFU of Daniel's strain of TMEV or phosphate-buffered saline (PBS) intracortically, 2 mm deep in the right hemisphere of the posterior parietal cortex, under isoflurane anesthesia and compressed air (Stewart et al., 2010a; Loewen et al., 2016; Patel et al., 2017). Mice were checked for handling-induced behavioral seizures twice a day, starting at 3 dpi as previously described. The seizures were scored based on a modified Racine scale: Stage 3, forelimb clonus; Stage 4, additional rearing; Stage 5, additional rearing and falling and few jumps; and Stage 6, additional clonic running, extensive jumping, falling, and severe hindlimb clonus (Racine, 1972; Patel et al., 2017). Only mice that have had at least one observed seizure above Stage 3 were used for experiments.

### Acute brain slice preparation

All in vitro experiments were performed during the peak period of TMEV infection (5 dpi) in the cortex, on the ipsilateral side of infection, in mice aged 10–20 weeks. At 5 dpi, mice were anesthetized using isoflurane anesthesia and oxygen. Once the mouse lost its righting reflex, it was rapidly decapitated; the brain was extracted and placed in ice-cold cutting *N*-methyl-d-glucamine (NMDG) solution for 10 s. The hindbrain was trimmed and was mounted on the vibratome (Vibratome 3000, Vibratome Company) with a back support of a 4% agarose (wt/vol) block while making sections. Coronal sections (350 μm) containing either the parietal or sensorimotor cortex and the hippocampus were made using the NMDG cutting solution (in mM: 92 NMDG, 2.5 KCl, 30 NaHCO_3_, 1.2 NaH_2_PO_4_, 20 HEPES, 25 glucose, 5 sodium ascorbate, 2 thiourea and 3 sodium pyruvate, 0.5 CaCl_2_, 10 MgSO_4_). Sections were incubated at 32–33°C for 30 min with regular 2,000 mM NaCl spike-ins depending on the age of the mouse (Ting et al., 2018). Subsequently, slices were transferred to room temperature (RT) HEPES holding solution (in mM: 92 NaCl, 2.5 KCl, 30 NaHCO_3_, 1.2 NaH_2_PO_4_, 20 HEPES, 25 glucose, 5 sodium ascorbate, 2 thiourea and 3 sodium pyruvate, 2 CaCl_2_,10 MgSO_4_) and held until imaging. All solutions were constantly bubbled with carbogen (95% O_2_/5% CO_2_) and pH titrated to 7.35 ± 0.05 and osmolarity at 290–310 mOsm. The reagents used for solution preparation were purchased from Sigma-Aldrich. The slices were imaged for up to 6 h after sectioning.

### Two-photon calcium imaging in microglia

Brain slices were placed in a custom slice chamber and hold-down (W4 64-0249, Warner Instruments) to prevent movement during imaging. Slices were continuously perfused with artificial cerebrospinal fluid (aCSF; in mM: 126 NaCl, 3 KCl, 26 NaHCO_3_, 1.4 NaH_2_PO_4_, 10 glucose, 2 CaCl_2_,12 MgSO_4_) by a peristaltic pump and bubbled with carbogen (95% O_2_/5% CO_2_). The temperature of the bath was maintained at 24–26°C using an in-line heater (TC-324C, Warner Instruments). Two-photon (2-P) calcium imaging was performed on a Prairie Ultima system (Bruker Corporation) using a Mai Tai DeepSee EHP 1040 laser (Spectra Physics) at 69 mW laser power, Prairie View software, a 20× water-immersion lens (NA: 1, Olympus), and emission bandpass filter at 560 nm to split green from red wavelengths (Bruker 370A510816). GCaMP6f and tdTom constitute the green and red channels, respectively, and images of microglia were acquired at 920 nm excitation, where both fluorophores were excited optimally. Microglial calcium imaging was performed ∼50 µm from the surface of the slice to exclude tissue damage due to slicing. ATP and ADP working solutions were prepared from stocks [10 mM ATP (Tocris Bioscience 3245)/ADP (Tocris Bioscience 1624) in reverse osmosis (RO) water, stored at −80°C]. The stocks were diluted to 100 µM ATP or ADP in aCSF with 15 µg/ml Alexa Fluor 568 (Invitrogen A33081) to visualize the puff. Puff pipettes were pulled by a HEKA PIP 6 electrode puller from 1.5 mm OD, thin-walled borosilicate glass and had an open tip resistance of 2–3.5 MΩ. ATP was dispensed using a Picospritzer III system (Parker Instrumentation) with 6 PSI pressure for 350 ms. The ipsilateral side receiving the TMEV or PBS injection was imaged in the cortical regions of layers II–V and either ATP or ADP was applied to different fields of view in the same brain slice. Time series images were acquired at 920 nm excitation, 2 Hz, 1.2 µs/pixel dwell, 512 × 512 pixels per frame, 2.5× optical zoom, and 240 pockels laser power for a 13 s baseline and 1 min after the puff.

### Detection of changes in calcium responses in response to ATP/ADP application

**Figure 2.**
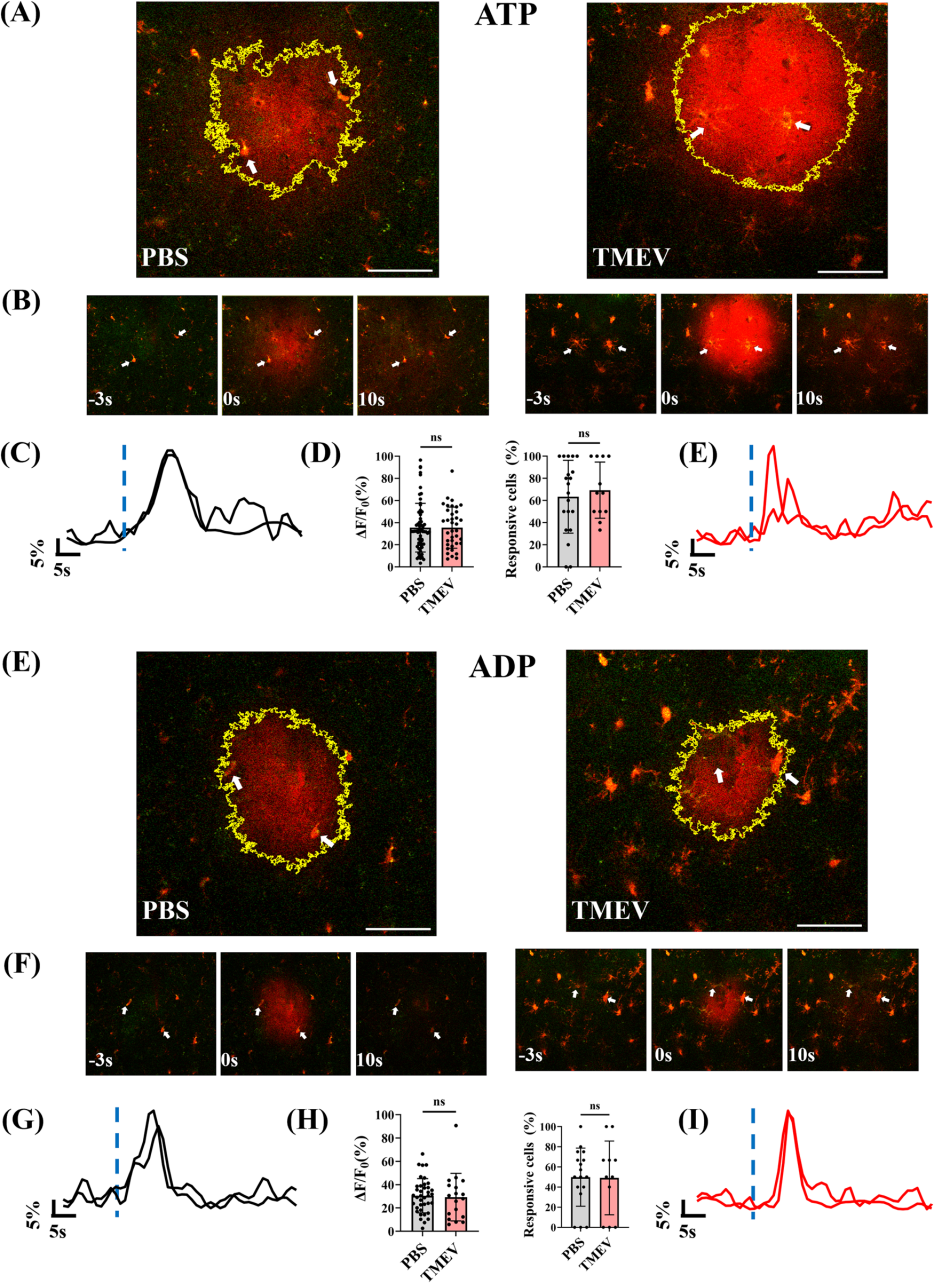
Microglial calcium responses to exogenous application of damage signals (ATP, ADP) remain unchanged as compared with PBS controls, at 5 dpi following TMEV infection in the cortex. ***A***, GCaMP6f-expressing microglia respond to an extracellular application of 100 μM ATP in acute brain slices from PBS (left panel) and TMEV mice (right panel). The highlighted yellow area indicates the spread of the ATP puff. Inclusion of Alexa Fluor 568 in the puffer pipette, while having spectral overlap with tdTom, allowed for identification of the spread of the agonist application. ***B***, Images showing the imaging field of view at pre- (−3 s), during (0 s), and postpuff for PBS and TMEV. White arrows are indicative of example microglia in the given field of view. ***C***, ***E***, Representative Δ*F*/*F*_0_ traces of PBS (black) and TMEV (red) of the microglia pointed out in panel ***B***. Blue-dashed vertical line indicating time of puff. ***D***, Bar graphs showing maximum Δ*F*/*F*_0_ changed and the percentage of cells exhibiting a calcium transient following agonist application across PBS and TMEV. PBS, *n* = 6 mice, 12 slices, 95 microglia/cells. TMEV, *n* = 4 mice, 7 slices, 56 microglia/cells. ***E***, GCaMP6f-expressing microglia respond to an extracellular application of 100 μM ADP in acute brain slices from PBS (left panel) and TMEV mice (right panel) with a fluorescent transient. The highlighted yellow area indicates the spread of the ADP puff. ***F***, Images showing the imaging field of view at pre- (−3 s), during (0 s), and postpuff for PBS and TMEV. White arrows are indicative of example microglia in the given field of view. ***G***, ***I***, Sample Δ*F*/*F*_0_ traces of PBS (black) and TMEV (red) of the microglia pointed out in panel ***F***. Blue-dashed vertical line indicating time of puff. ***H***, Bar graphs showing maximum Δ*F*/*F*_0_ changed and the percentage of cells exhibiting a calcium transient following agonist application across PBS and TMEV. PBS, *n* = 6 mice, 11 slices, 72 microglia/cells. TMEV, *n* = 4 mice, 8 slices, 40 microglia/cells. Independent sample *t* test or Mann–Whitney test were applied based on normality testing (Shapiro–Wilk test). Scale bar, 50 µm.

To quantify changes in microglial calcium response to ATP and ADP puffs, we reduced the image noise with a hybrid 3D median filter in ImageJ (Schindelin et al., 2015). The area of ATP agonist spread in the brain slice was identified by the spread of Alexa Fluor 568 in the postapplication period, as described in Umpierre et al. (2019). Of note, Alexa Fluor 568 has a spectral overlap with the TdTomato, and thus, in images, the puff radius appears red. Next, the area of the puff was demarcated, and a mask was created in order to facilitate detection of cells within the puff. Using CellProfiler (Stirling et al., 2021) cell image analysis software, various microglial cells were identified and segmented, rendering them as regions of interest (ROIs). This output was further analyzed on ImageJ, and measures of the ROIs were calculated from the mean pixel intensity in the ROI (F) for each point in time. Microglia inside the puff diameter (representative region demarcated in Fig. 2*A*,*E*) were analyzed to keep the measurements consistent across slices. The microglia outside the puff diameter were excluded from the analysis. Using a custom MATLAB (MathWorks) script, the maximum of calcium signal change (*F* − *F*_0_)/*F*_0_ was calculated compared with the mean pixel intensity for baseline 13 s before the application (*F*_0_). The baseline was defined as the mean of the fluorescent intensity values before the puff. The event threshold is set to two standard deviations above the initial baseline fluorescence and the “findpeaks” feature was used. Δ*F*/*F*_0_ time series plots were generated by averaging *F* values of each pixel at each time point and using the mean fluorescence of all image frames as the baseline fluorescence (*F*_0_).

### Cytokine and chemokine panel

TMEV-infected and PBS-control mice (aged 12–16 weeks) were killed at 7 dpi by transcardial perfusion with PBS, and the ipsilateral hippocampi and cortices were dissected and flash-frozen in liquid nitrogen. The tissue was homogenized using a mechanical homogenizer in ∼50 μl 1× PBS. Furthermore, the tissue was lysed with a lysis buffer (R&D Systems 895347) and protease inhibitor cocktail (Roche, 04693159001), centrifuged, following which the lysate was extracted for protein quantification using the BCA assay kit (Thermo Fisher Scientific 23225). Normalized protein amounts across samples were loaded to perform the cytokine assay. Cytokine and chemokine levels were measured using the LEGENDplex Mouse Inflammation Panel (BioLegend 740446), according to manufacturer's protocol. Briefly, standards were prepared by serially diluting standards provided in the kit (used for calibration of analyte curves). Assay buffer (25 μl) and samples/standards (25 μl) were added to wells of a 96-well V-bottom plate. A 25 μl of mixed beads was added after vortexing them (to avoid bead settling). Samples were shaken at 80 rpm on a plate shaker for 2 h at RT and centrifuged at 1,050 rpm for 5 min, and the supernatant was discarded in one continuous and forceful motion. The centrifugation step was repeated once more, and 25 μl biotinylated detection antibodies were added to each well, followed by shaking at 800 rpm for 1 h at RT. A 25 μl of streptavidin-PE (SA-PE) was added to each sample and incubated for 30 min. Finally, samples were washed with the wash buffer, and the beads were resuspended. Samples were subjected to flow cytometry analysis (BD CytoFlex) on the same day as the assay. Data analysis was performed using BioLegend's LEGENDplex data analysis software, and statistical analysis was carried out on GraphPad Prism (version 9.4).

### RNAscope in situ hybridization and immunohistochemistry

TMEV-infected and PBS-control mice (aged 10–12 weeks) were killed at 5 dpi using excessive isoflurane and transcardially perfused with 1× PBS briefly for ∼30 s (until the liver was ∼75% clear to preserve RNA quality), followed by 10% neutral buffered formalin solution (NBF). The brains were postfixed for 24 h in 10% NBF and subsequently transferred to a 15/30% sucrose gradient for cryoprotection. Coronal sections of the brain were made (15 µm thickness) using a freezing stage microtome (Leica SM 2010R). Duplicate sections from each brain were mounted on Superfrost slides (Thermo Fisher Scientific, 1255015) and processed for RNAscope (controls included). Fluorescent in situ hybridization (FISH) was performed as per the manufacturer's instructions using RNAscope Multiplex Fluorescent Reagent Kit v2 for Fixed Frozen Tissue using catalog probes TNF-α (catalog #311081), P2Y_12_R (catalog #317601-C2) and P2X_7_R (catalog #316311-C2).

Briefly, brain sections were heated for 30 min at 60°C in a Rototherm Mini Plus (Benchmark Scientific H2024) and postfixed in prechilled 10% NBF for 15 min at 4°C. The tissue was then dehydrated by running it through a 50, 70 and 100% ethanol gradient for 5 min each and then air-dried. Subsequently, after the addition of RNAscope hydrogen peroxide (incubation for 10 min) and washes, target retrieval was performed by boiling the slides for ∼7 min in a Bella Food steamer with the target retrieval agent. Afterward, a hydrophobic barrier was applied (ImmEdge, Vector Laboratories H-4000), following which sections were incubated in Protease III for 30 min at 40°C in a water bath. Probe hybridization (2 h) was performed, followed by hybridization of AMPs and Opal Dyes 520 and 690. Slides were washed twice each, with wash buffer and 1× PBS subsequently, and immunohistochemistry was then performed.

Brain sections were stained with an IBA1 (ionized calcium-binding adaptor molecule 1) primary antibody (microglia/macrophage marker, 1:500 dilution, Novus Biologicals NB100-1028) overnight at 4°C, in 0.5% Triton X-100 (Sigma-Aldrich, T8787) on an orbital shaker. The following morning, after two washes with PBS, slides were stained with secondary antibody Alexa Fluor donkey anti-goat 546 for 2 h, at RT on the orbital shaker. Furthermore, slides were counterstained with DAPI (Advanced Cell Diagnostics) and mounted with Prolong Gold antifade reagent (Molecular Probes) to image on Leica TCS SP8 X White Light Laser Confocal Microscope with a 40×/1.3 oil CS2 objective. Microscope settings including laser intensity, photomultiplier, and offset were optimized to yield the highest signal-to-noise ratio, to reduce saturated pixels across samples, and, once finalized, the parameters were held constant between samples. Five ROIs (1 µm *z*-stack each) in the cortex were imaged in every mouse brain slice, mounted as duplicates. Positive (PPIB) and negative (dapB) control probes were used to confirm the specificity of the probes.

#### Quantification/data analysis

*z*-stack images were first preprocessed on the Imaris software (version 10.1.0; Bitplane AG), using a Gaussian filter. Using the “Spots” feature on Imaris, spots were generated for FISH (RNAscope) probes (refer to Extended Data Fig. 4-1 for settings). Using the “Cell feature” and customized settings, IBA1^+^ cells were delineated. RNA “Spots” were imported into the “Cell” feature as vesicles, and all quantification of “Spots” and “Cells” were exported using the Imaris “statistics” feature. To have an unbiased approach, the settings for “Spots” for various RNAscope channels were finalized without enabling the IBA1 channel and vice versa. The “Spots” settings were held consistent between TMEV and PBS brain sections. The parameters for defining “Cells” in the TMEV and PBS conditions varied slightly (Extended Data Fig. 4-1) due to changes in IBA1^+^ expression (Loewen et al., 2016).

### Statistics

Using GraphPad Prism version 10.1.2, based on normality analysis, for comparisons between two groups, Student's *t* test (two-tailed) or nonparametric, Mann–Whitney test was used. Data are represented as mean ± standard deviation (STD), and a *p* value of <0.05 was considered statistically significant.

## Results

Mice (10–20 weeks) were intracranially injected with TMEV or PBS and monitored and scored twice a day from Days 3–7 postinjection for handling-induced behavioral seizures. Only mice in the TMEV treatment group that had a seizure above Grade 3 on the Racine scale were used for subsequent experiments. The timeline for TAM administration, TMEV injection, seizure monitoring, and subsequent experiments is shown in Figure 1.

### Reactive microglia respond to exogenous application of damage signals following TMEV infection (5 dpi) in the cortex

In TMEV-infected mice, seizures generalize and spread secondarily into the cortex following TMEV infection (Patel et al., 2017). Additionally, microglia in the parietal cortex following infection become reactive (Loewen et al., 2016; Bell et al., 2020). It has been demonstrated that reactive microglia in the hippocampus of TMEV-infected mice have a diminished calcium response to exogenous application of ATP and ADP, and this is likely due to the decreased expression of P2Y_12_R observed following infection (DePaula-Silva et al., 2019; Wallis et al., 2024). ADP is the primary ligand which binds to P2Y_12_R. ATP signaling has also been shown to lead to cationic influx (including Na^+^, Ca^2+^) through the ionotropic purinergic receptor, P2X_7_R (mechanistic biomarker of epilepsy), and evokes extrusion of K^+^ currents, which in turn has been shown to trigger apoptotic cascades via the NLRP3 inflammasome (Muñoz-Planillo et al., 2013; Di Virgilio et al., 2017). Therefore, we hypothesized that reactive cortical microglia would also have diminished calcium responses following exogenous application of ATP and ADP.

In order to evaluate changes in microglial responses to purinergic damage signals in the parietal cortex as a result of TMEV infection, either ATP (100 µM) or ADP (100 µM) was applied extracellularly to acute brain slices while imaging microglia in mice expressing tdTomato and GCaMP6f. Figure 2*A* shows panels of the field of view in the respective brain slices (PBS and TMEV) at the instance of the ATP puff. Figure 2*B* shows time instances corresponding to pre-, during, and postpuff time points, and white arrows indicate examples of microglia taken into consideration for calcium fluorescence analysis. The changes in Δ*F*/*F*_0_ of GCaMP6f fluorescence are shown in PBS (Fig. 2*C*) and TMEV (Fig. 2*E*) conditions. Upon puffing 100 μM ATP in brain slices from PBS-injected and TMEV-infected mice, despite the presence of reactive microglia in the cortex following TMEV infection, their calcium response (Δ*F*/*F*_0_) to ATP was unaltered at the peak period of TMEV infection, at 5 dpi (Fig. 2*D*). Similarly, Figure 2*E* shows panels of the field of view in the respective brain slices (PBS and TMEV) following the ADP puff. Figure 2*F* shows panels elucidating pre-, during, and post-ADP puff, and panels *G* and *I* are representative Δ*F*/*F*_0_ traces of microglia as indicated by white arrows in panel *F*. The calcium responses of TMEV-infected mice microglia were comparable to those of PBS controls upon 100 μM ADP puffing (Fig. 2*H*). These results, along with glial reactivity profiles from previous studies (Loewen et al., 2016; Bell et al., 2020), suggest that even though there is microglial reactivity in the cortex of TMEV mice, they are able to respond to purinergic damage signals, comparable to PBS controls.

### Differential proinflammatory cytokine and chemokine responses in the cortices and hippocampi of TMEV mice

**Figure 3.**
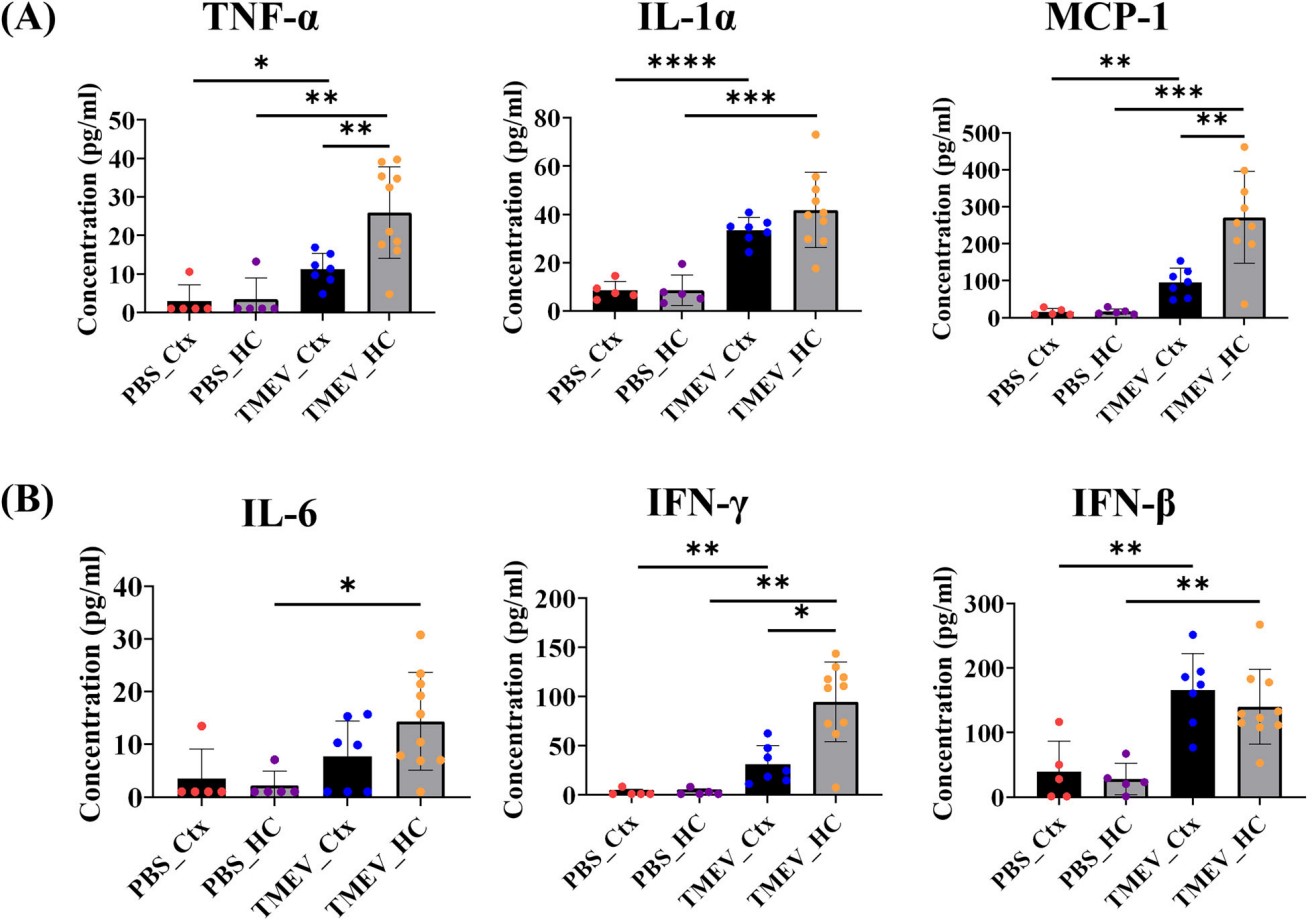
Significant increase in protein levels of inflammatory markers in the cortex and hippocampus of TMEV-infected mice during the acute seizure phase. ***A***, TNF-α and the chemokine MCP-1 are increased significantly in TMEV, as compared with PBS controls in both the cortex and hippocampus. IL-1α is increased because of TMEV and comparable in the TMEV-infected cortex and hippocampus. ***B***, IL-6 is significantly increased in the hippocampus in TMEV, and IFN-γ and IFN-β are increased in the hippocampus and cortex as a result of TMEV infection, as compared with PBS controls. *n* = 5 mice (PBS), 10 mice (TMEV). Independent sample *t* test or Mann–Whitney test were applied based on normality testing (Shapiro–Wilk test). ***p < 0.001; **p < 0.01; *p < 0.05. Refer to Extended Data Figure 3-1 for additional cytokines.

In TMEV-infected mice, microglia and macrophages contribute to a cascade of innate immune responses in the CNS, initiating the generation of a cytokine storm encompassing high levels of production of proconvulsant cytokines like TNF-α, IL-1β, IL-6, and IFN-γ and chemokines (Cusick et al., 2013; DePaula-Silva et al., 2021). In order to explore brain region-based differences in cytokine and chemokine profiles as a result of TMEV infection, a comprehensive predefined inflammation panel (LEGENDplex Mouse Inflammation Panel, BioLegend 740446) was conducted to assess protein levels. We found a significant increase in the proinflammatory cytokines TNF-α and IL-1α (produced by macrophages and monocytes, which can further activate TNF-α), and the chemokine MCP-1/CCL2 (regulator of migration and infiltration of monocytes/macrophages) in the cortex of TMEV-infected mice, as compared with PBS controls (Fig. 3*A*; compare TMEV_Ctx vs PBS_Ctx). These levels were further increased in the hippocampus (Fig. 3*A*; compare TMEV_HC vs TMEV_Ctx) which represents the primary site of TMEV damage possibly due to TMEV tropism for the pyramidal neurons in this area (Stewart et al., 2010a,b). Additionally, we found a significant increase in levels of the proinflammatory/proconvulsant cytokine IL-6 in the hippocampus of TMEV-infected mice, as previously described (Fig. 3*B*).

10.1523/ENEURO.0374-25.2026.f3-1 Figure 3-1 **Significant decreases in certain cytokine levels in the hippocampus, due to TMEV infection, during the acute seizure phase.** Protein levels of IL-23, IL-1β and IL-27 were significantly decreased in the hippocampus of TMEV-infected mice, as compared to PBS controls. n = 5 mice (PBS), 10 mice (TMEV). Independent samples t-test or Mann-Whitney test were applied based on normality testing (Shapiro-Wilkins test). **p<0.01, *p<0.05. Download Figure 3-1, DOCX file.

We also found that the levels of IFN-γ, which is involved in the antiviral response (with ability to inhibit viral replication) and IFN-β, (which reduces excessive neuroinflammation) were increased in both the hippocampus and cortex of TMEV-infected mice compared with PBS controls (Fig. 3*B*). The levels of IL-1α (Fig. 3*A*) and IFN-β (Fig. 3*B*) between the hippocampus and cortex of TMEV-infected mice were comparable for these cytokines. Additionally, we also observed a reduction in levels of IL-23 (inflammatory cytokine for T helper cell maintenance and expansion), IL-1β (produced by activated macrophages, mediator of inflammatory responses), and IL-27 (may have pro- or anti-inflammatory responses depending on local cues and can lead to IL-10 expression) in the hippocampus in TMEV as compared with PBS controls (Extended Data Fig. 3-1). Overall, for the first time, a gradation of changes in the cytokine and chemokine profiles of cortices and hippocampi were observed in TMEV mice, and in general, there were higher levels of proinflammatory cytokines in the hippocampi > cortices > PBS controls. This suggests that there is a heterogeneity of cellular reactivity profiles with respect to pro- and anti-inflammatory cytokines and chemokines as a result of TMEV infection in brain regions crucial for studying viral infection-induced TLE in mice.

### Increases in purinergic receptor expression and TNF-α following TMEV infection in cortical microglia

**Figure 4.**
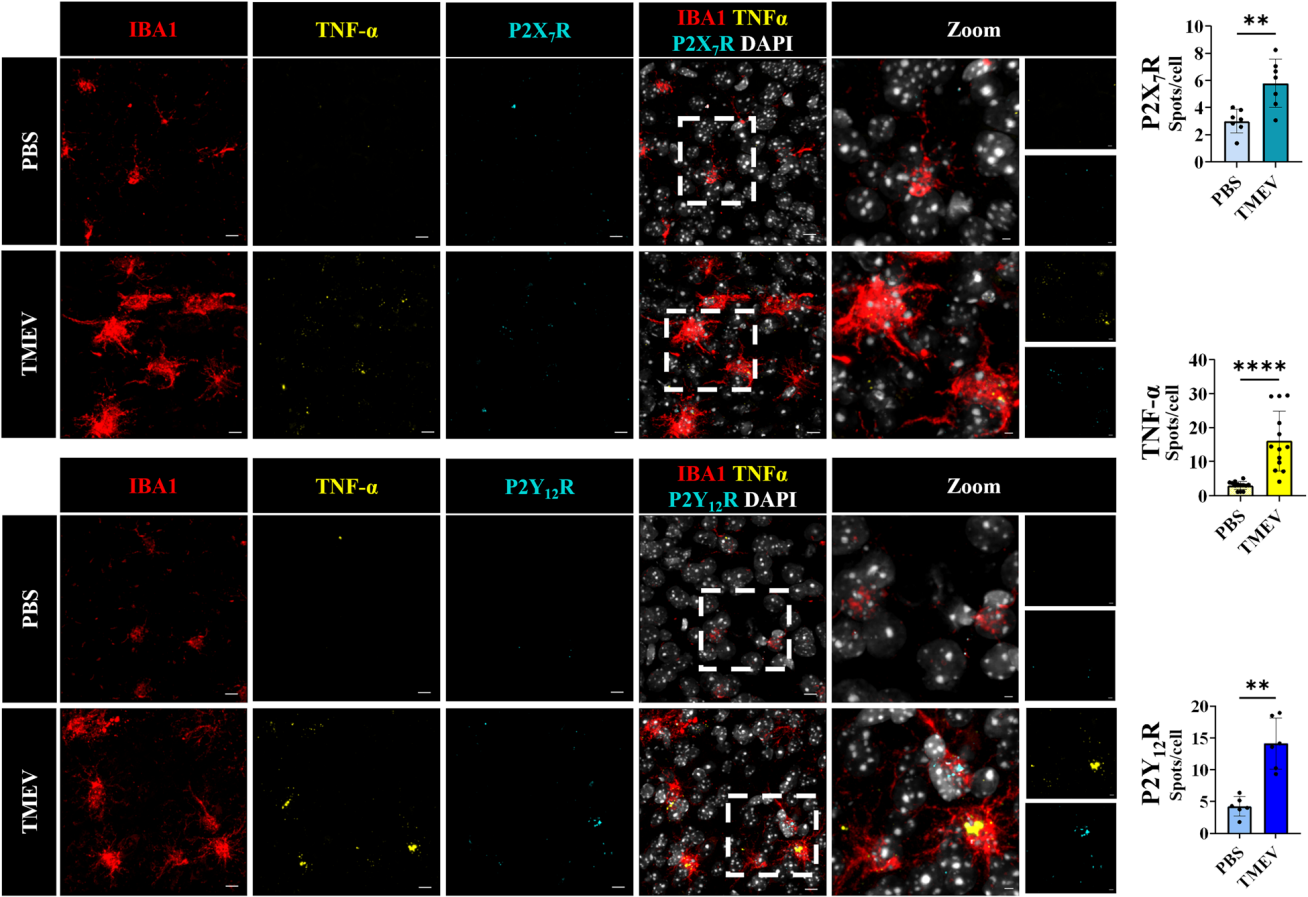
Significant increases in microglial RNA levels of purinergic receptors and TNFα as a result of TMEV infection (acute phase). Increased colocalization of TNFα and P2X_7_R (in situ hybridization; top panel) and TNFα and P2Y_12_R mRNA expression (bottom panel) in IBA1-positive cells (IHC) in the cortex of 5 d post-TMEV infection. Mice, P2X_7_R, *n* = 4 per condition (PBS/TMEV); sections, 2 per mouse; P2Y_12_R, *n* = 3 per condition (PBS/TMEV); sections, 2 per mouse; TNFα, *n* = 7 per condition (PBS/TMEV); sections, 2 per mouse. Independent samples *t* test or Mann–Whitney test were applied based on normality testing (Shapiro–Wilk test). *p < 0.05; **p < 0.01; ***p < 0.001; ****p < 0.0001. Scale bar, 10 µm; for zoomed sections, 3 µm. Refer to Extended Data Figure 4-1 for analysis parameters.

In order to study a few downstream receptors of ADP and ATP, we evaluated the purinergic receptor expression of P2Y_12_R and P2X_7_R, along with the inflammatory cytokine TNF-α as a positive control in microglia using RNAscope in situ hybridization and immunohistochemistry to quantify RNA puncta in IBA1^+^ cells. P2X_7_R has been shown to be a biomarker of epilepsy; its hyperactivation has been observed in various disorders and plays a crucial role in amplifying CNS damage in neurodegenerative diseases (Ribeiro et al., 2021; Engel, 2023). P2Y_12_R plays a critical role in microglial homeostasis, in microglial process extension and retraction (Gómez Morillas et al., 2021). The number of P2X_7_R, TNF-α, and P2Y_12_R RNA puncta was significantly increased in IBA1^+^ cells in the cortex as a result of TMEV infection at 5 dpi (Fig. 4). Collectively, this indicated that there was upregulation of proinflammatory markers like TNF-α in the cortex, also observed in the hippocampus (Patel et al., 2017; Hanak et al., 2019; Wallis et al., 2024). This is also consistent with the calcium transient responses of microglia to application of purinergic agonists, as observed using calcium imaging in our slice experiments, wherein microglia have comparable magnitudes of calcium transients to agonist application in the cortex following TMEV infection and comparable to that observed in slices obtained from PBS controls. However, P2Y_12_R expression in the cortex is in contrast with what was observed in the hippocampus of TMEV-infected mice during the peak period of infection, where lower expression was concurrent with lower motility to damage signals and a reduced magnitude of agonist-induced calcium transients (Wallis et al., 2024).

10.1523/ENEURO.0374-25.2026.f4-1 figure 4-1 **Parameters for RNAScope analysis.** Table listing all analysis parameters used for spot and cell detection on Imaris software for RNAScope analysis. Download figure 4-1, DOCX file.

## Discussion

In this study, we used a multidisciplinary approach to investigate brain region-based changes in microglial reactivity profiles following TMEV infection, at the RNA, cytokine (protein), and cellular calcium imaging scales/levels. We demonstrated that post-TMEV infection, cortical microglia retain their ability to respond to localized purinergic insults (ADP/ATP), comparable to PBS controls, despite being reactive, in acute brain slice preparations. We also found that the levels of several pro- (TNF-α, IL-1α, IL-6, and IFN-γ) and anti-inflammatory cytokine (IFN-β) are brain region dependent in TMEV-infected mice, with most increasing in the order of PBS controls, cortices, and hippocampi of TMEV mice. We also observed increases in expression of purinergic receptors crucial for microglial motility (P2Y_12_R) and inflammation (P2X_7_R) in the cortex using RNAscope FISH. Several studies have demonstrated that there is a decreased expression of P2Y_12_R in the acute seizure phase (Haynes et al., 2006; Alves et al., 2017; DePaula-Silva et al., 2019); thus the increase we observed in the cortical microglia was novel, especially given that reactive hippocampal microglia have been shown to have a dramatic reduction in this receptor in the same model of TMEV infection (Wallis et al., 2024). Overall, following TMEV infection, significant region-based differences demonstrate that reactive cortical microglia retain their ability to respond to purinergic damage signals, the cortex has a higher production of proinflammatory cytokines, and there is an increased expression of certain purinergic receptors, as compared with PBS controls.

Microglia are immune sentinels in the brain and form an integral functional unit in the CNS via the “quad-partite synapse” (Schafer et al., 2013) with neurons and astrocytes. Microglia constantly survey the local environment in the brain and play critical roles in responding to CNS insults, brain damage repair, surveillance, neuroinflammation, and synaptic pruning (Nayak et al., 2012; Eyo et al., 2017). The dynamic cross talk between microglia and neurons is facilitated by fractalkine signaling, and soluble signaling molecules like ATP act as a “find-me” distress signal for microglial motility and process elongation (Eyo et al., 2014).

Microglial calcium signaling and its dysregulation is one of the hallmarks in various CNS disorders including Alzheimer's disease (AD), stroke, multiple sclerosis (MS), Parkinson's disease (PD), and epilepsy (Pan and Garaschuk, 2023; Hasan et al., 2024; Wang et al., 2024). Microglia can also adopt a plethora of morphological and functional phenotypes, but the two may not overlap (Brawek and Garaschuk, 2013; Walker et al., 2014). Following CNS insults/injuries and neuroinflammation, there is increased microglial calcium signaling observed, which could further orchestrate downstream second messenger cascades, implying their role in pathogen response and epileptogenesis (Pozner et al., 2015; Eyo et al., 2017; Riester et al., 2020; Umpierre et al., 2024).

Microglial calcium signaling is a crucial component of neuron–glia communication in health and modulates inflammatory and structural responses in disease. In epilepsy, there is an increase in microglial calcium transients during seizures and early epileptogenesis, driven by purinergic signaling, which couples hyperexcitability to microglial reactivity and structural remodeling (Umpierre et al., 2020). In AD, microglia near the amyloid beta plaques display an increase in frequency and intensity of spontaneous calcium transients in their processes, as compared with somata (Izquierdo et al., 2024). Additionally, there is mitochondrial dysfunction and apoptosis observed as a result of microglial calcium dysregulation (Calvo-Rodriguez and Bacskai, 2021). In PD, there is persistent microgliosis, release of proinflammatory cytokines, and elevated microglial calcium levels leading to mitochondrial dysfunction and acceleration of neurodegeneration (Hopp, 2021; Muzio et al., 2021). Due to the upregulation of microglial calcium influx, there are downstream implications to the NLRP3 inflammasome and neuroinflammation in MS (Zhang et al., 2021).

Particularly, P2Y_12_R is a G_i_-coupled GPCR, which when activated induces chemotaxis in microglia. ADP acts as a primary agonist for P2Y_12_R, while ATP, upon hydrolysis to ADP, can serve as an agonist as well. P2Y_12_R plays a crucial initiating role in microglial motility and migration toward CNS injury and surveillance and in extension and retraction of microglial processes (Haynes et al., 2006; Gómez Morillas et al., 2021). Increased expression of purinergic receptors like P2X_7_R and P2Y_12_R have been reported as a result of viral infections in the CNS and subsequent exacerbation due to production of proinflammatory cytokines (Alves et al., 2020). P2Y12R expression has been shown to decrease in the acute seizure phase and increased reactivity but increased after status epilepticus (Haynes et al., 2006; Alves et al., 2017; DePaula-Silva et al., 2019; Wang et al., 2023). Infected neurons have been shown to recruit P2Y_12_R-positive microglia during encephalitis in the human brain, and severity of the infection dictated the level of leukocytes and microglial reactivity/responses (Fekete et al., 2018). There is reduced microglia–neuron interaction in mice lacking P2Y_12_R, along with increased severity and lethality during status epilepticus (Eyo et al., 2014; Badimon et al., 2020). The implications of increases in P2Y_12_R are still under investigation, while certain groups have reported increases in P2Y_12_R due to viral infections like HIV, as summarized by Alves et al. (2020). Additionally, Irino et al. (2008) showed that increases in [Ca^2+^]_i_ by P2Y_12_ receptor-mediated PLC activation to be necessary for ADP-induced chemotaxis of microglia.

In a recent study, decreases in levels of P2Y_12_R and reduced microglial motility to injury (laser burn) were observed in the hippocampus in TMEV-infected mice (Wallis et al., 2024). There is considerable neuronal degeneration of CA1 pyramidal cells within the hippocampus of TMEV-infected mice and a downregulation of P2Y_12_R was also reported in bulk RNA-seq experiments by DePaula-Silva et al. (2019) as a result of TMEV. Interestingly, in the present study, we show that reactive cortical microglia in TMEV-infected mice have increased P2Y_12_R expression at the RNA level (Fig. 4) at the same time point postinfection that reactive microglia in the hippocampus exhibit a decreased expression (Wallis et al., 2024). The present experiments evaluating cytokine panels (Fig. 3) also corroborates the increases in expression of several proinflammatory cytokines including TNF-α, IFN-γ, and IL-1α, in the cortex of TMEV-infected mice versus PBS controls. Notably, the levels of some of these cytokines and the chemokine MCP-1 are further increased in the hippocampus, in accordance with previous studies from the lab detailing increased proinflammatory profiles in the hippocampus of TMEV mice (Loewen et al., 2016; Patel et al., 2017; Bell et al., 2020). It will be important to assess microglial motility in the cortex following TMEV infection to determine if microglia in the cortex retain their motility. Given that there is an increase in the mRNA expression of P2Y_12_R in the cortex and robust purinergic responses to agonist applications, we would hypothesize that motility is also unaffected. The therapeutic value of P2Y_12_R (Chen et al., 2019) also implores further assessment of implications of its beneficial effect in the cortex, as observed in our results in TMEV-infected mice.

Our data show an upregulation of P2X_7_R at the RNA level, at the peak period of TMEV infection (5 dpi) in the cortex, as compared with age-matched PBS controls. This further defines the reactivity of IBA1^+^ cells in the cortex of TMEV mice, but further evaluation at various extended time points (like 14 dpi) could shed light on the evolution/reduction of these reactivity profiles. The P2X_4_ receptor is also stimulated by ATP in microglia and is involved in chemotaxis along with P2Y12_R_ (Ohsawa et al., 2007). Another crucial purinergic receptor to be investigated in TMEV is the P2Y_6_ receptor. UDP activation of P2Y_6_R leads to microglial phagocytosis of dying cells in epilepsy (Umpierre et al., 2024), and future studies could help unravel the phagocytic changes in purinergic signaling as a result of TMEV infection across the cortex and hippocampus.

P2X_7_R, an ionotropic purinergic receptor, has been shown to be a mechanistic biomarker in epilepsy, and other conditions like AD and COVID-19 (Illes, 2020; Ribeiro et al., 2021; Engel, 2023). As a result of ATP signaling, the P2X_7_ receptor allows for an influx of cations, including Ca^2+^ and Na^+^, and evokes extrusion of K^+^ currents. P2X_7_R signaling is also crucial to the NLRP3 inflammasome-mediated triggering of apoptotic cascades, leading to cell inflammation and cell death (Di Virgilio et al., 2017). Furthermore, microglial plasma membrane blebbing can be a consequence of ATP-induced P2X_7_R activation (Illes, 2020). Potassium efflux has been shown to trigger activation of caspases and inflammasome activation via the P2X_7_ receptor (Muñoz-Planillo et al., 2013). Recently, it has been discovered that K^+^ efflux also mediates P2Y_12_R-dependant inflammasome activation (Suzuki et al., 2020), thereby unraveling novel roles of these receptors. A recent study by Mitlasóczki et al. showed that in TMEV-infected mice, following seizure activity, there were spreading depolarizations in the hippocampus but not the neocortex (Mitlasóczki et al., 2025). Additionally, Patel et al. recently showed that increased expression of the ECM component chondroitin sulfate proteoglycans in the dentate gyrus and amygdala could be one of the causal factors for TMEV-induced seizures (Patel et al., 2024). These studies further reiterate the importance of delving into region-based differences in infection-induced epilepsy.

In conclusion, we evaluated region-specific differences in cytokine profiles and determined the microglial purinergic receptor-mediated calcium imaging responses in the cortex in the TMEV model of infection-induced TLE. Despite microglia being reactive in the cortex (confirmed by P2X_7_R increases, phenotypic morphology, and cortical increases in cytokine expression), there are no changes in the calcium signaling response to application of ATP or ADP following infection, in stark contrast to that observed in the hippocampus (Wallis et al., 2024). Future work investigating (1) different time points, like 2 dpi (before seizures begin in the TMEV model) and 14 dpi (after seizures resolve and the virus is cleared in the TMEV model), (2) microglial motility in the cortex of TMEV-infected mice, and (3) an in-depth study of other purinergic receptors using tools like spatial transcriptomics could unravel more information about the evolution of the neuroinflammatory states in cortical microglia as a result of TMEV infection.

## Data Availability

All data generated or analyzed during this study are included in this published article (and its Extended Data files).

## Synthesis

Reviewing Editor: Harald Sontheimer, University of Virginia Decisions are customarily a result of the Reviewing Editor and the peer reviewers coming together and discussing their recommendations until a consensus is reached. When revisions are invited, a fact-based synthesis statement explaining their decision and outlining what is needed to prepare a revision will be listed below. The following reviewer(s) agreed to reveal their identity: Bhanu Tewari.

The reviewers found yourstudy to be clear and straightforward, anmd found the findings not unexpected and reflecting microglial responses that are brain-region specific. A more expanded discussion of the novelty of the study should be included. Authors can consider assessing the microglial motility or whether and how the expression further changes during the course of epileptogenesis, as they mentioned. OR any other relevant question that brings a novel conclusion to this study.

Several specific improvements are recommend as follows:

1) The study mentions that both male and female mice were used, but it's unclear how sex was handled statistically. Could there be more explanation of sex-specific analysis?

2) The conclusion that cortical microglia "retain responsiveness" could be clarified further. What does this mean biologically for epileptogenesis?

3) In Figure 2, which microglia were analyzed? Those inside the puff, or a combination of inside and outside? It would be interesting to see how microglial response varies with increasing distance from the puff, as concentrations will be lower farther from the puff's epicenter.

4) Some of the reviews cited are over 20 years old. They are therefore obsolete (i.e., Engel 2001, line 61, among others). Given that our understanding of microglia has increased substantially over the last 10 years, I recommend updating the cited literature.

5) The introduction is rather long and loses focus.

6) The statement on IACUC guidelines needs a reference (line 134). See PMID: 32663221.

7.) There is not enough background information in the introduction on the role of microglial calcium signaling and how it is important in homeostasis and diseases. Also, what area of the cerebral cortex was used in the study?

8.) In the Ca imaging images, despite using a GECI, the background appears unusually high, especially after ATP application in Fig. 2A. Can the authors clarify this? Also, Fig. 1D and H are missing the data points in the bar graphs.

9.) The representative traces of Ca response in C, E, G, and I are fine; however, the authors should ideally add an average trace with the grayed-out background traces of individual cells.

10.) The pseudocolor intensity scale should be added in all relevant places (see PMID: 32716294, Fig. 3C and F for reference).

## Author Response

Synthesis Statement for Author (Required):

The reviewers found your study to be clear and straightforward, and found the findings not unexpected and reflecting microglial responses that are brain-region specific. Therefore a more expanded discussion of the novelty of the study should be included. Authors can consider assessing the microglial motility or whether and how the expression further changes during the course of epileptogenesis, as they mentioned. OR any other relevant question that brings a novel conclusion to this study.

We appreciate that the reviewers found our study clear and straightforward. We have edited the introduction and conclusion to more clearly indicate the novel findings of our study. Given that P2Y12R expression in reactive microglia is generally decreased during the acute seizure phase, the finding that reactive cortical microglia had increased expression of P2Y12R was novel. This is in stark contrast to what was observed in the dorsal hippocampus from TMEV-treated mice (Wallis et al., 2024), where decreased expression of P2Y12R, as well as considerably attenuated calcium transients in response to purinergic agonist application, were observed in reactive microglia. This regional diversity in the function of reactive microglia was unexpected, as neuroinflammation is present in both regions and importantly, seizures propagate from the hippocampus to the cortex. We have highlighted these novel findings in the introduction and discussion.

Several other specific improvements are recommended as follows:

1) The study mentions that both male and female mice were used, but it's unclear how sex was handled statistically. Could there be more explanation of sex-specific analysis? Thank you for pointing this out. The data for the experiments from both sexes of mice were pooled, since there is no suggestion of sex specific differences in seizure behavior in the TMEV mouse model (Patel et al., 2017). We have now mentioned this in the methods section of the manuscript.

2) The conclusion that cortical microglia "retain responsiveness" could be clarified further. What does this mean biologically for epileptogenesis? We appreciate that the phrase 'responsiveness' is vague. To address this, throughout the manuscript, we have been more precise in our language, and the discussion has been expanded to address the biological ramifications for seizures.

3) In Figure 2, which microglia were analyzed? Those inside the puff, or a combination of inside and outside? It would be interesting to see how microglial response varies with increasing distance from the puff, as concentrations will be lower farther from the puff's epicenter.

Thank you for giving us an opportunity to clarify. We analyzed the microglia inside the puff diameter (puff region is demarcated in Figure 2 A and E) to keep the measurements consistent across slices. The microglia outside the puff diameter were excluded from the analysis, as back calculation of agonist concentration in those regions might not be accurate. We have added information about this in the methods section of the manuscript.

4) Some of the reviews cited are over 20 years old. They are therefore obsolete (i.e., Engel 2001, line 61, among others). Given that our understanding of microglia has increased substantially over the last 10 years, I recommend updating the cited literature.

Thank you, we have updated/ added certain recent references. We also have cited certain legacy papers in the field along with updated references from recent years (2020 onwards). The references also include those entailing the progression and results in the TMEV model in the Wilcox/ Fujinami labs in the past decade until updated manuscripts.

5) The introduction is rather long and loses focus.

Thank you, we have shortened the introduction, updated references, and modified the sections to be succinct.

6) The statement on IACUC guidelines needs a reference (line 134). See PMID: 32663221.

Thank you, we have added references and links for this in the manuscript.

7) There is not enough background information in the introduction on the role of microglial calcium signaling and how it is important in homeostasis and diseases. Also, what area of the cerebral cortex was used in the study? We appreciate this criticism and have added a short write up about microglial calcium signaling and its role in health and disease in the introduction and a short paragraph in the discussion section. Also, the sensorimotor and parietal cortex areas of the cerebral cortex, at the level of the hippocampus, were used in this study and this is now specifically mentioned in several places throughout the manuscript.

8) In the Ca imaging images, despite using a GECI, the background appears unusually high, especially after ATP application in Fig. 2A. Can the authors clarify this? Also, Fig. 1D and H are missing the data points in the bar graphs.

Thank you for giving us an opportunity to clarify about this. We used Alexa 568 as the dye to visualize the puff for downstream analysis. The ATP and ADP were prepared with the Alexa dye, which falls into the red spectrum. This has a spectral overlap with the tdTomato; therefore, the visualization of the puff is in the same color as tdTomato. The central snapshot panels (Figure 2A and E) show the field of view during the puff (Alexa 568 dye), which is red, and is not the background of tdTomato expression. Also, we added the data points in Fig 2D and H in the manuscript. The impact of using Alexa 568 has now been more carefully explained in the methods and figure legend of figure 2.

9) The representative traces of Ca response in C, E, G, and I are fine; however, the authors should ideally add an average trace with the grayed-out background traces of individual cells.

Thank you for the suggestion, we have added data points to the bar graphs in Figure 2 (D and H). However, the images shown are not averages, but are the actual traces (and thus representative) from the microglia indicated by arrows in the image.

10) The pseudocolor intensity scale should be added in all relevant places (see PMID: 32716294, Fig. 3C and F for reference).

Thank you for the suggestion about the pseudocolor intensity. Pseudocolor intensity scales as mentioned in the PMID reference usually are used when the data is represented as raster plots/ heatmaps (as shown in PMID reference). Since we are showing direct snapshots of the field of view under the microscope for calcium imaging at various time points under the 2P microscope and representative traces, it would not be accurate to add a pseudo color intensity scale. We measure ΔF/F0 in the manuscript, and have not used ratiometric imaging, which is generally illustrated using pseudo color intensity scales as raster plots/ heatmaps.

REFERENCES:

Patel, D. C., Wallis, G., Dahle, E. J., McElroy, P. B., Thomson, K. E., Tesi, R. J., Szymkowski, D. E., West, P. J., Smeal, R. M., &Patel, M. (2017). Hippocampal TNFα signaling contributes to seizure generation in an infection-induced mouse model of limbic epilepsy. Eneuro, 4(2).

Wallis, G. J., Bell, L. A., Wagner, J. N., Buxton, L., Balachandar, L., &Wilcox, K. S. (2024). Reactive microglia fail to respond to environmental damage signals in a viral-induced mouse model of temporal lobe epilepsy. bioRxiv, 2024.2003. 2006.583768.
