# Supplementary material for: Investigating the Role of Cortical Microglia in a Mouse Model of Viral Infection-Induced Seizures
Source: eNeuro. 2026 Feb 24;13(2):ENEURO.0374-25.2026. doi: 10.1523/ENEURO.0374-25.2026 (PMC12931998; doi:10.1523/ENEURO.0374-25.2026)
Supplement: Figure 3-1 — Significant decreases in certain cytokine levels in the hippocampus, due to TMEV infection, during the acute seizure phase. Protein levels of IL-23, IL-1β and IL-27 were significantly decreased in the hippocampus of TMEV-infected mice, as compared to PBS controls. n = 5 mice (PBS), 10 mice (TMEV). Independent samples t-test or Mann-Whitney test were applied based on normality testing (Shapiro-Wilkins test). **p<0.01, *p<0.05. Download Figure 3-1, DOCX file. [file eneuro-13-ENEURO.0374-25.2026-s002.docx]

**Extended Data Figure 3-1. Significant decreases in certain cytokine levels in the hippocampus, due to TMEV infection, during the acute seizure phase.** Protein levels of IL-23, IL-1β and IL-27 were significantly decreased in the hippocampus of TMEV-infected mice, as compared to PBS controls. n = 5 mice (PBS), 10 mice (TMEV). Independent samples t-test or Mann-Whitney test were applied based on normality testing (Shapiro-Wilkins test). **p<0.01, *p<0.05.
