# Supplementary figures and images for: Investigating the Role of Cortical Microglia in a Mouse Model of Viral Infection-Induced Seizures
Source: eNeuro. 2026 Feb 24;13(2):ENEURO.0374-25.2026. doi: 10.1523/ENEURO.0374-25.2026 (PMC12931998; doi:10.1523/ENEURO.0374-25.2026)

**Extended Data Figure 4-1: Parameters used for RNAScope analysis**


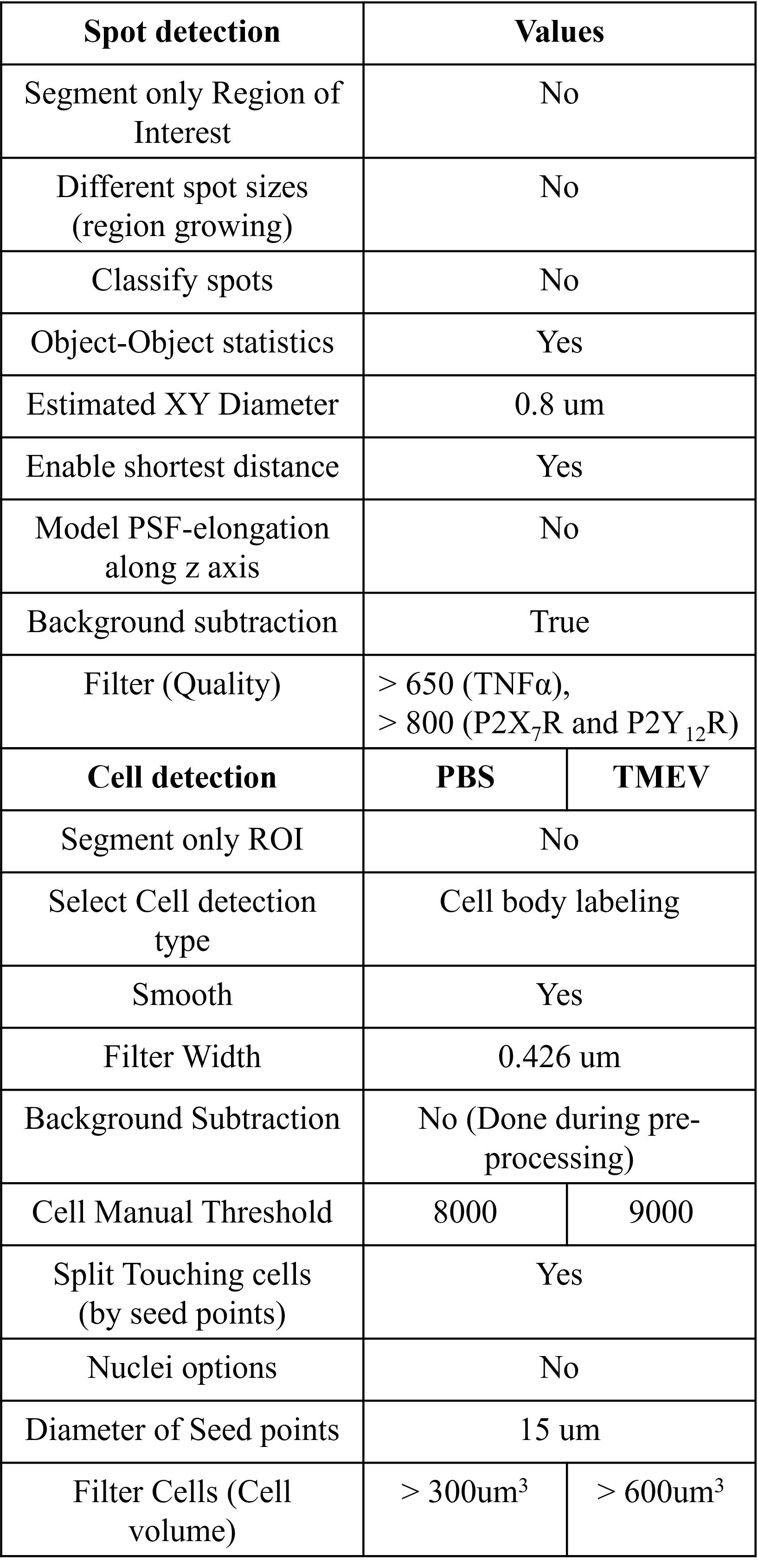

Supplement: figure 4-1 — Parameters for RNAScope analysis. Table listing all analysis parameters used for spot and cell detection on Imaris software for RNAScope analysis. Download figure 4-1, DOCX file. [file eneuro-13-ENEURO.0374-25.2026-s003.docx]
